# Identification of a prognostic index system and tumor immune infiltration characterization for lung adenocarcinoma based on mRNA molecular of pyroptosis

**DOI:** 10.3389/fmed.2022.934835

**Published:** 2022-09-15

**Authors:** Huawei Li, Xiaoyan Chang, Haiyan Wang, Bo Peng, Jun Wang, Pengfei Zhang, Linyou Zhang

**Affiliations:** ^1^Department of Thoracic Surgery, The Second Affiliated Hospital of Harbin Medical University, Harbin, Heilongjiang, China; ^2^Department of Pediatrics, 83 Group Military Hospital of People’s Liberation Army, Xinxiang, Henan, China

**Keywords:** lung adenocarcinoma, pyroptosis, immunotherapy, PRMPI, OS

## Abstract

**Background and purpose:**

Pyroptosis is a form of programmed cell death, which plays an important role in tumorigenesis, progression, and regulation of the tumor microenvironment. It can affect lung adenocarcinoma (LUAD) progression. This study aimed to construct a pyroptosis-related mRNA prognostic index (PRMPI) for LUAD and clarify the tumor microenvironment infiltration characterization of LUAD.

**Materials and methods:**

We performed a univariate Cox regression analysis for pyroptosis-related mRNAs in the TCGA cohort. Then, we used LASSO Cox regression to establish a PRMPI. The quantitative real time polymerase chain reaction (qRT-PCR) was used to quantify the relative expression of pyroptosis-related mRNAs. The CPTAC cohort was used to confirm the stability and wide applicability of the PRMPI. The single-sample gene set enrichment analysis (ssGSEA) was performed to assess the tumor microenvironment infiltration characterization.

**Results:**

A total of 36 pyroptosis-related mRNAs were identified. The PRMPI was established based on five pyroptosis-related mRNAs. The expression patterns of these mRNAs were verified in LUAD samples from our medical center by qRT-PCR. High-PRMPI patients had worse overall survival than low-PRMPI patients. The result was validated in the CPTAC cohort. The comprehensive analysis indicated that the high-PRMPI patients exhibited lower immune activity, more aggressive immunophenotype, lower expression of immune checkpoint molecule, higher TP53 mutation rate, and higher tumor stemness than low-PRMPI patients. Low-PRMPI patients may be more sensitive to immunotherapy, while high-PRMPI patients may benefit more from chemotherapy and targeted therapy.

**Conclusions:**

The PRMPI may be a promising biomarker to predict the prognosis, tumor microenvironment infiltration characterization, and the response to adjuvant therapy in LUAD.

## Introduction

Lung cancer is a leading cause of cancer-related deaths worldwide and approximately half of these cancers are lung cancer (1). Since most patients with lung cancer are initially diagnosed at an advanced stage, their 5-year survival rate is only about 18%, even if the diagnosis and treatment have been greatly improved (2). The main treatment methods of lung adenocarcinoma (LUAD) include surgery, radiotherapy, chemotherapy, targeted therapy, and immunotherapy. Immunotherapy, which activates the immune system of patients to suppress tumor cells, has significantly prolonged the survival of patients with lung cancer (3). Since there are undetectable T cells in the tumor microenvironment could release some cytokines, which became refractory to PD-1 inhibitors (4). Therefore, only a small number of patients with advanced lung cancer received durable responses and improved survival from immunotherapy, so it is urgent to improve the overall disease control rate (5). There are many immunosuppressive cells and immunosuppressive molecules related to the tumor immune escape in the lung cancer microenvironment (4). Therefore, it is necessary to further explore the characteristics of the LUAD transcriptome to construct a gene signature that can accurately predict the prognosis and efficacy of adjuvant therapy (such as immunotherapy and targeted therapy).

Pyroptosis is a type of programmed cell death (PCD), which is caused by the formation of pores or breaches in the cell membrane (6). During lytic cell death, the destruction of cellular integrity is caused by water inflow, loss of membrane potential, and cell swelling, which eventually lead to cell rupture (6). Consequently, the cellular contents are released, which promote the release of pro-inflammatory cytokines by stimulating macrophages or other bystander cells, leading to a strong inflammatory response (6). Pyroptosis is a double-edged sword, which can not only form a microenvironment to promote tumor cell growth but also inhibit tumor growth by promoting cell death (7). Recent studies have found that echinacoside can exert its anti-tumor effects by inducing pyroptosis in non-small-cell lung cancer (8). The induction of pyroptosis, ferroptosis, and necroptosis of tumor cells can alter the tumor microenvironment (TME) and promote the influx of tumor-infiltrating lymphocytes in small-cell lung cancer, and the combination of their inducers and immune checkpoint inhibitors can enhance the anti-tumor effects (9). It is reasonable to speculate that pyroptosis may be closely related to the regulation of TME in LUAD. Unfortunately, we know little about the relationship between pyroptosis and TME of LUAD, and effective prognostic prediction and indicators of adjuvant therapy are desperately needed.

In our study, we constructed a pyroptosis-related mRNA prognostic index (PRMPI) in the TCGA cohort using five differentially expressed pyroptosis-related mRNAs (DEPRMs), and the reliability and stability of PRMPI were validated in the CPTAC cohort. To verify our findings more effectively, we quantified the expression levels of these five DEPRMs by using the quantitative real time polymerase chain reaction (qRT-PCR) method in 49 pairs of LUAD tissues and adjacent non-tumorous tissue obtained from our hospital. Afterward, we comprehensively explored the relationship between the PRMPI and the characterization of tumor immune infiltration, expression of immune checkpoint molecule, immune score, tumor stemness, and tumor mutation state. Moreover, we also sought to investigate the association between the PRMPI and the sensitivity of patients with LUAD to immunotherapy, chemotherapy, and targeted therapy.

## Materials and methods

### Lung adenocarcinoma sample collection, RNA extraction, and quantitative real time polymerase chain reaction

A total of 49 LUAD and corresponding paracancerous tissue samples (Table 1) were collected from the Second Affiliated Hospital of Harbin Medical University. Ethics approval was granted by the Ethics Committee in the Second Affiliated Hospital of Harbin Medical University (Ethics Approval Document No. KY2021-286).

**TABLE 1:** Clinical characteristics of 49 LUAD patients.

Characteristics	Median (IQR)/No. of LUAD patients
Age	62 (57–68)
**Gender**	
Male	22
Female	27
**Grade**	
Low- differentiation	14
Moderate- differentiation	15
Well- differentiation	12
Unknown	8
**T stage**	
Tis	1
T1	38
T2	7
T3	3
**N stage**	
N0	45
N1	3
N2	1
**TNM stage**	
I	42
II	6
III	1

LUAD, lung adenocarcinoma; IQR: inter quartile range.

According to the manufacturer protocol, total RNA was extracted from LUAD tissues and corresponding paracancerous tissues using RNA Extraction Reagent (Vazyme Biotech, China). The total RNA (1 μg) was reverse-transcribed into complementary DNA (cDNA) using a HiScript^®^ II 1st Strand cDNA Synthesis Kit (Vazyme Biotech, China). The ChamQ SYBR qPCR Master Mix (Vazyme Biotech, China) was used for qRT-PCR. The primer sequence was designed and synthesized by GENERAL BIOL (Anhui, China) and was shown in Supplementary Table 1. GAPDH was used for normalization. The relative expression level of these genes was calculated by using the 2^–ΔΔCT^ method. Then, we compared the relative expression levels of the gene in tumor and normal tissue using the Wilcoxon matched-pairs signed rank test by R software.

### Datasets

**FIGURE 1:**
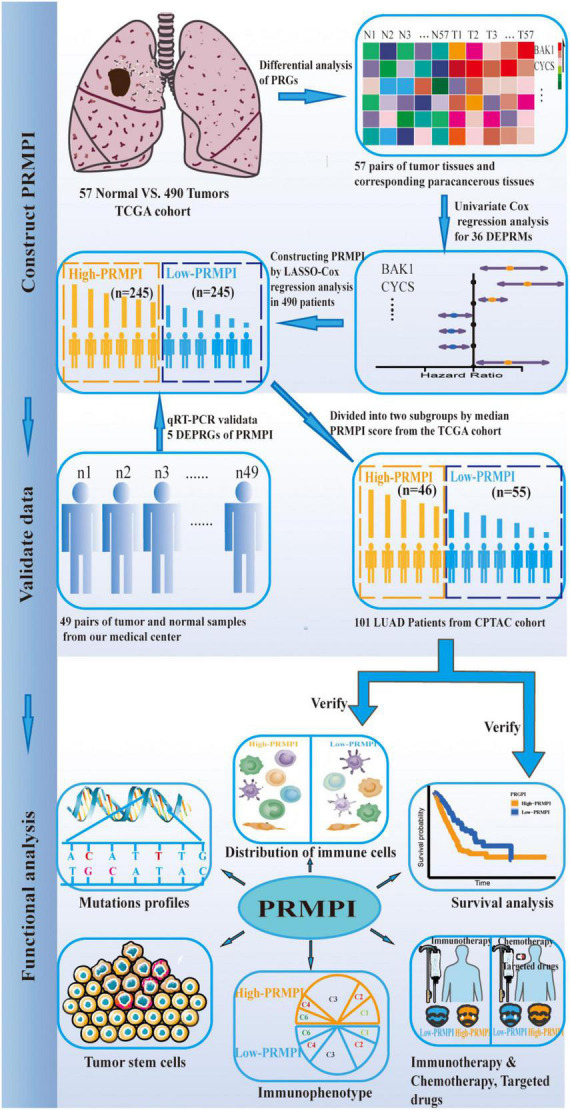
Flow diagram of this study.

The workflow diagram of this study is shown in Figure 1. In The Cancer Genome Atlas (TCGA)^1^ database, there are 516 LUAD samples and 59 paracancerous samples with RNA sequencing data, of which 57 LUAD samples correspond to 57 paracancerous samples. In addition, there are 559 patients with LUAD with masked somatic mutation data and 522 patients with LUAD with clinical information in the TCGA database. Therefore, RNA-Seq (FPKM) data, masked somatic mutation data, and clinical information were downloaded from the TCGA database. In the clinical proteomic tumor analysis consortium (CPTAC, see text footnote 1) database, there are 111 LUAD samples and 102 paracancerous samples with RNA sequencing data, of which 111 LUAD samples serve as the validation dataset. These RNA-seq (FPKM) data were downloaded from the CPTAC program of the GDC Data Portal, and the clinical information was obtained from the CPTAC Data Portal.^2^

For survival analysis and Cox regression analysis, the inclusion criteria of patients are as follows: a. with complete clinicopathological information, such as age, gender, TNM stage, and histopathological classification; b. follow-up or survival time of more than 30 days; c. RNA-seq data are available. The exclusion criteria are as follows: a. follow-up or death within 30 days, or the follow-up status unknown; b. age, gender, and TNM stage are unknown; d. no RNA-seq data. Finally, we performed further survival analysis for 490 patients with LUAD in the TCGA cohort and 101 patients with LUAD in the CPTAC cohort (Supplementary Figure 1). Before the survival evaluation, we used the “ComBat” function in the “sva” package to correct the batch effect of TCGA and CPTAC datasets from non-biological technical bias. In addition, based on previous publications (7, 10–16), expression matrixes of 56 pyrolysis-related mRNAs (PRMs) were extracted from TCGA (Supplementary Table 2).

### Construction and validation of pyroptosis-related mRNA prognostic index system

Differentially expressed pyroptosis-related mRNAs (DEPRMs) were identified using the Wilcoxon signed-rank test in 57 pairs of LUAD tissues and corresponding paracancerous non-tumor tissues with a false discovery rate (FDR) of <0.05 in TCGA cohort. Univariate Cox regression analysis was performed with the “survival” package in R. Then, the PRMPI was established using the least absolute shrinkage and selection operator (LASSO) Cox-regression analysis with the “glmnet” package for DEPRMs with prognostic significance in the TCGA cohort (17). The optimal penalty parameter that was estimated by using 10-fold cross-validation was used to screen gene signatures. The PRMPI score formula was constructed by using the coefficient of each gene calculated by LASSO regression and the expression level of each gene. The formula was used to calculate the PRMPI score for each patient with LUAD in the TCGA cohort and CPTAC cohort, and the patients were divided into the high-PRMPI score and low-PRMPI score subgroups based on the median PRMPI score from the TCGA cohort. The principal component analysis (PCA) and t-distributed stochastic neighbor embedding (t-SNE) based on five PRM signatures were performed with the “Rtsne” package to explore the distribution of high-PRMPI and low-PRMPI subgroups in the two cohorts. The prognostic power of the PRMPI was analyzed by using Kaplan–Meier (K–M) survival analysis method with log-rank tests in two subgroups. The time-dependent ROC curve analysis was implemented with the “timeROC” and “survival” packages to evaluate the prognostic value of the PRMPI. Furthermore, to explore the independent prognostic significance of the PRMPI, we performed univariate and multivariate Cox analyses on the pathological stage, gender, age, and PRMPI in TCGA and the CPTAC cohorts.

### Comprehensive analysis of tumor microenvironment infiltration characterization in different pyroptosis-related mRNA prognostic index subgroups

We analyzed 29 immune-associated gene sets, which represented diverse immune cell types, functions, and pathways. The single-sample gene set enrichment analysis (ssGSEA) was used to quantify the activity or enrichment scores of immune cells, functions, or pathways in each tumor sample based on immune-associated gene sets. Then, we compared the GSVA enrichment scores of 16 immune cells and 13 immune-related pathways between high-PRMPI and low-PRMPI subgroups by using the Wilcoxon signed-rank test, and the *p*-value was adjusted by using the BH method (18). In addition, we also compared the expression levels of immune checkpoint molecules in the two PRMPI subgroups by using the Wilcoxon signed-rank test. The immune score that reflects the level of immune cell infiltration was calculated for each patient with LUAD using the “estimate” package (19). The correlation between the PRMPI score and immune checkpoint molecule and immune score were analyzed by using the Spearman correlation test. The relationship between the PRMPI score and six immune subtypes (20) was tested by two-way ANOVA. Categorical variables were analyzed by using the Chi-square test.

The DNA methylation-based stemness index (DNAsi) and mRNA expression-based stemness index (mRNAsi) of TCGA LUAD cases were used to assess cancer stem cell (CSC) features in LUAD (21). Then, we used the Spearman correlation analysis to analyze the association of the PRMPI score with tumor stemness. The landscape plot of tumor mutation profiles in PRMPI subgroups was presented by using the “waterfall” function of the “maftools” package.

### Predict response of patients with lung adenocarcinoma to immunotherapy

We downloaded immunophenoscore (IPS) data of patients with LUAD from The Cancer Immunome Atlas (TCIA) database, which is positively correlated with cancer immunogenicity and can predict the response of patients with cancer to immunotherapy (18). We compared the IPS between two PRMPI subgroups by using the Wilcoxon signed-rank test, and the results were shown by using the violin plot.

### Evaluation of the significance of pyroptosis-related mRNA prognostic index score in chemotherapy and targeted therapy

To evaluate the clinical value of the PRMPI score in LUAD chemotherapy and targeted therapy, we converted the TCGA gene expression matrix into the half inhibitory centration (IC50) data matrix of the corresponding anti-tumor drugs by “pRRophetic” package and then analyzed the IC50 difference between the high-PRMPI and low-PRMPI subgroups by using the Wilcoxon signed-rank test, and the results were shown by a bar chart.

## Results

### Identification of differentially expressed pyroptosis-related mRNAs

**FIGURE 2:**
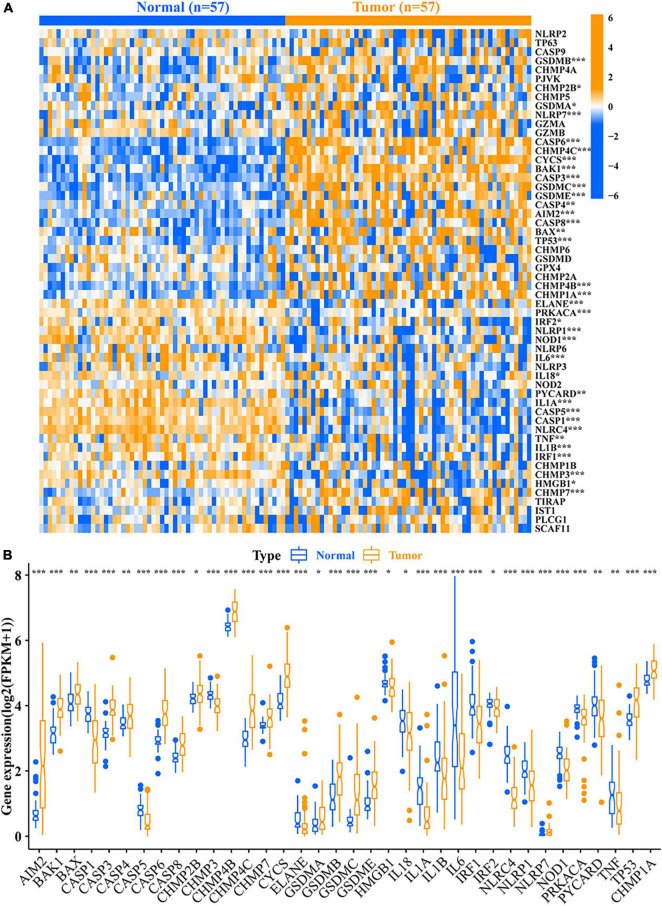
Relative expression level of 56 PRMs. **(A)** Heat map of the PRMs between the normal tissues and the tumor tissues. **(B)** Expression level of 36 DEPRMs in the normal and tumor tissues. *p-value < 0.05; **p-value < 0.01; and ***p-value < 0.001. PRMs, pyroptosis-related mRNAs; DEPRMs, differentially expressed pyroptosis-related mRNAs.

A total of 36 DEPRMs was identified in 57 pairs of LUAD tissues and paracancerous tissues. Among them, 21 genes were upregulated, while 15 genes were downregulated in the LUAD tissues (Figures 2A,B).

### Development and validation of pyroptosis-related mRNA prognostic index system

**FIGURE 3:**
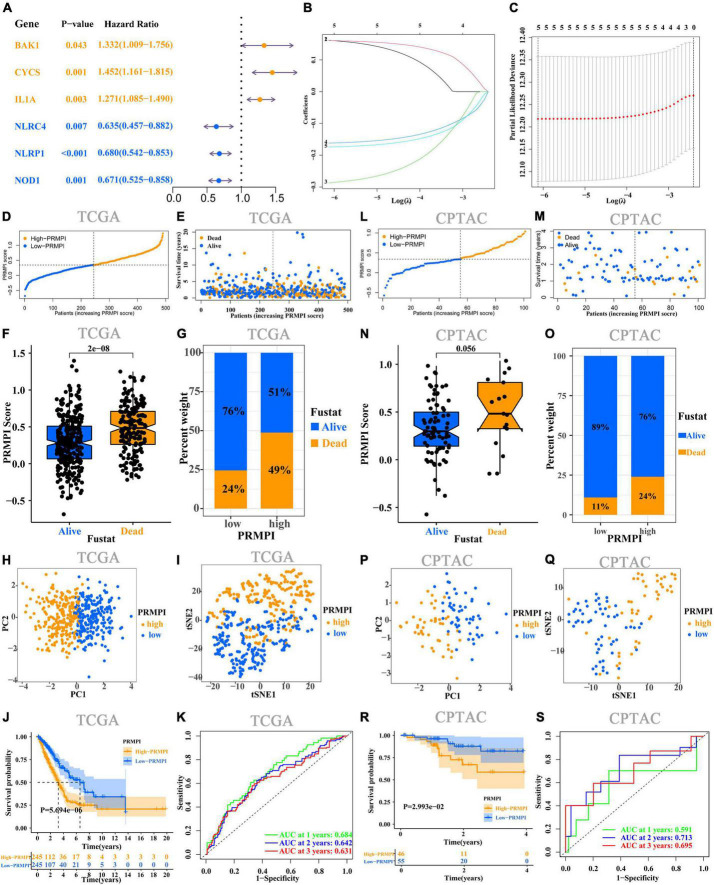
Construction and prognostic analysis of PRMPI. **(A)** Univariate Cox regression analysis for 36 DEPRMs. **(B)** LASSO Cox regression coefficients of five DEPRMs with prognostic significance. **(C)** Cross-validation of tuning parameter selection in the LASSO Cox regression. **(D)** Distribution of patients with LUAD based on the PRMPI score. **(E)** Follow-up time and survival status for each patient with LUAD. **(F)** Comparation of PRMPI score between alive and dead LUAD patients. **(G)** Proportion of alive and dead patients in two PRMPI subgroups. PCA plot **(H)** and t-SNE analysis **(I)**. **(J)** Kaplan–Meier survival analysis of the low-PRMPI and high-PRMPI subgroups. **(K)** ROC analysis of PRMPI scores on OS at 1-, 2-, and 3-year follow-ups. **(L–S)** Relationship between the PRMPI and survival was validated by RNA-seq and clinical data from the CPTAC cohort. PRMPI, pyroptosis-related mRNA prognostic index; DEPRMs, differentially expressed pyroptosis-related mRNAs; PRMs, pyroptosis-related mRNAs; LUAD, lung adenocarcinoma; OS, overall survival; ROC, receiver operating characteristics.

Univariate Cox regression analysis was performed on the 36 DEPRMs, and six DEPRMs with prognostic significance were identified (Figure 3A). However, the expression level of IL1A in LUAD was significantly lower than that in paracancerous tissues, but survival analysis showed that its high expression was associated with a worse prognosis, so we removed IL1A in further survival analysis.

According to the minimum standard of LASSO regression, five DEPRMs were selected to construct the formula of the PRMPI score based on the regression coefficient and expression level of five DEPRMs [PRMPI score = (0.1619 × BAKI expression level) + (0.1614 × CYCS expression level)−(0.2872 × NLRC4 expression level)−(0.1617 × NLRP1 expression level)−(0.1741 × NOD1 expression level)] (Figures 3B,C). All patients with LUAD in the TCGA cohort were divided into low- and high-PRMPI subgroups according to the median PRMPI score (Figure 3D). The survival status plots indicated that the number of deaths in the high-PRMPI subgroup was higher than that in the low-PRMPI subgroup (Figures 3E–G). PCA and t-SNE analyses were performed for the expression level of five DEPRMs and PRMPI scores. The results showed that the patients in the low- and high-PRMPI subgroups were distributed in two different directions (Figures 3H,I). Consistently, the Kaplan–Meier (K–M) curves showed that patients with LUAD with a higher PRMPI have a shorter OS than those with a lower PRMPI in the TCGA cohort (Figure 3J).

The ROC curve confirmed that the PRMPI has moderately predictive potential for OS (AUC = 0.631 at 3-years, AUC = 0.642 at 2-years; AUC = 0.684 at 1-years, Figure 3K).

To evaluate the stability of the PRMPI, according to the median PRMPI score from the TCGA cohort, we also divided patients with LUAD into low- and high-PRMPI score subgroups in the CPTAC cohort. As with TCGA results, there were more deaths in patients in the high-PRMPI score subgroup than in the low-PRMPI score subgroup (Figures 3L–O). Similar to the TCGA cohort, t-SNE and PCA analyses for these five genes and PRMPI score showed a discrete distribution of patients with LUAD in low- and high-PRMPI score subgroups (Figures 3P,Q). Moreover, the result of the K-M survival analysis for patients with LUAD in the CPTAC cohort was similar to that of patients in the TCGA cohort (Figure 3R). In addition, the AUC of the PRMPI was 0.695 at 3 years, 0.713 at 2 years, and 0.591 at 1 year (Figure 3S). These results showed that the PRMPI has excellent stability and wide applicability.

### Verification of the five differentially expressed pyroptosis-related mRNAs of the pyroptosis-related mRNA prognostic index system

**FIGURE 4:**
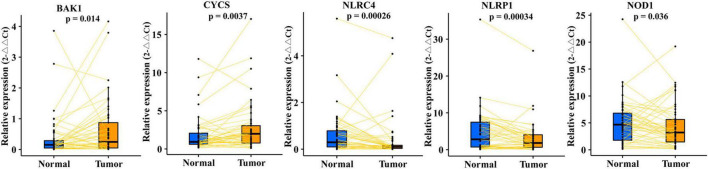
qRT-PCR confirmed five differentially expressed PRMs constructing the PRMPI model between LUAD and adjacent non-tumor tissues. PRMPI, pyroptosis-related mRNA prognostic index; PRMs, pyroptosis-related mRNAs; LUAD, lung adenocarcinoma.

To verify the five differentially expressed genes of PRMPI (BAK1, CYCS, NLRC4, NLRP1, and NOD1) between tumor and normal tissues, qRT-PCR was performed to quantify the gene expression. Our results showed that BAK1 and CYCS were upregulated, and NLRC4, NLRP1, and NOD1 were downregulated in LUAD tissues compared with paracancerous tissues (Figure 4, *p*-value < 0.05).

### Pyroptosis-related mRNA prognostic index was an independent predictor of overall survival for patients with lung adenocarcinoma

**FIGURE 5:**
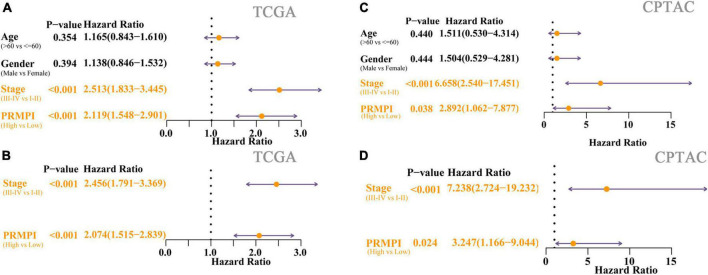
Cox regression analyses for the clinicopathological factors and PRMPI scores. **(A)** Univariate Cox regression analysis of clinicopathological and the PRMPI score for TCGA cohort. **(B)** Multivariate Cox regression analysis of the factors that have statistical differences in the univariate analysis (*p*-value < 0.05) TCGA cohort. **(C)** Univariate Cox regression for the CPTAC cohort. **(D)** Multivariate Cox regression analysis for the CPTAC cohort. OS, overall survival; PRMPI, pyroptosis-related mRNA prognostic index.

Univariate Cox regression analysis showed the high-PRMPI score and advanced TNM stage (Figure 5A) were related to a worse prognosis. Multivariate Cox regression analysis indicated that a high PRMPI (HR: 2.074, 95% CI: 1.515-2.839, *p* < 0.001) and advanced TNM stage (HR: 2.456, 95% CI: 1.791–3.369, *p* < 0.001) were independent prognostic factors for patients with LUAD in TCGA cohort after correcting for other confounding factors (Figure 5B). These results were validated by the CPTAC cohort (Figures 5C,D).

### Tumor microenvironment infiltration characterization

Since pyroptosis can affect the TME and the body’s response to immunity (22–24), we further explored the relationship between the PRMPI constructed by five PRMs and the tumor microenvironment infiltration characterization.

**FIGURE 6:**
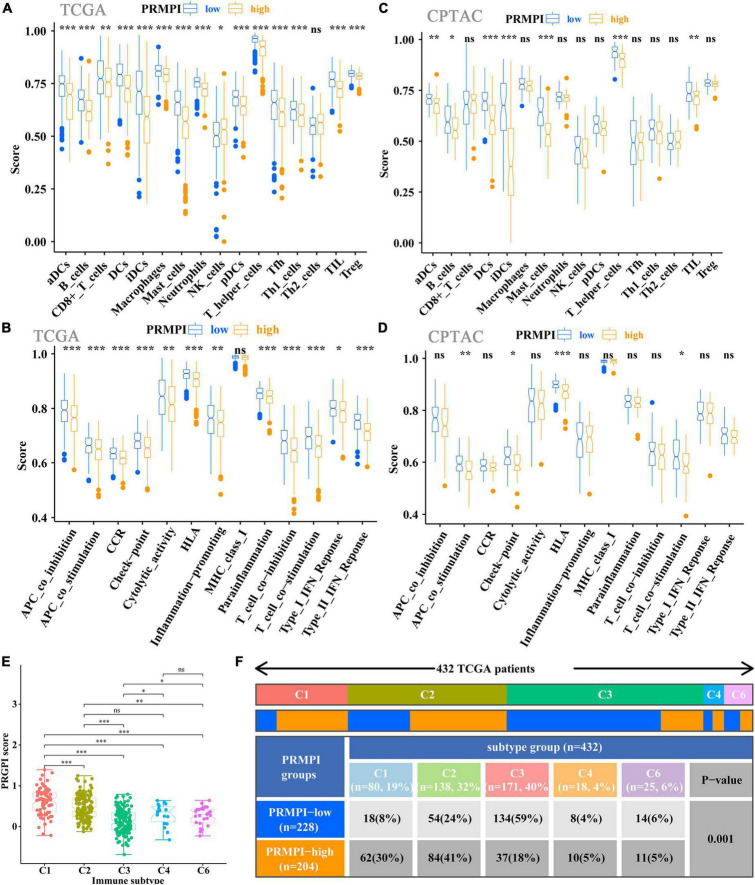
Tumor-infiltrating immune cells and immune-related pathways between the PRMPI subgroups. **(A,B)** Enrichment scores of 16 types of tumor-infiltrating immune cells and 13 immune-related pathways between the PRMPI subgroups in TCGA cohort. **(C,D)** Enrichment scores of 16 types of tumor-infiltrating immune cells and 13 immune-related pathways between the PRMPI subgroups in the CPTAC cohort. **(E)** Comparison of the PRMPI score in five immunophenotypes (C1, C2, C3, C4, C6). **(F)** Heat map and table showing the distribution of five immunophenotypes between the PRMPI subgroups. The distributions of the immunophenotype in the PRMPI subgroups were compared by using the chi-square test. *p-value < 0.05; **p-value < 0.01; ***p-value < 0.001. PRMPI, pyroptosis-related mRNA prognostic index. ns, no significance.

The enrichment scores of 16 immune cells and 13 immune-related pathways in the high- and low-PRMPI subgroups were quantified by using ssGSEA in TCGA and CPTAC cohorts. Except for NK cells, the infiltration level of the other 14 immune cells in the high-PRMPI subgroup was significantly lower than that in the low-PRMPI subgroup in the TCGA cohort (Figure 6A). Among the 13 immune-related pathways, except for the MHC-I pathway, the activity of the other 12 immune pathways in the high-PRMPI subgroup was lower than that in the low-PRMPI subgroup (Figure 6B). To verify this result, we further evaluated the tumor immune status of LUAD in the CPTAC cohort and found that the immune status in the high- and low-PRMPI subgroups were similar to that in the TCGA cohort (Figures 6C,D).

To understand how the PRMPI score is related to the cytotoxic components of tumor immune infiltration, we analyzed the relationship between the PRMPI score and six immunophenotypes: wound healing (C1), IFN-γ-dominant (C2), inflammatory (C3), lymphocyte-depleted (C4), immunologically quiet (C5), and TGF-β-dominant (C6). The results showed that 432 patients with LUAD were classified into five immune subtypes in the TCGA cohort. Since no patient with LUAD was assigned to the C5 immunophenotype subgroup, C5 was excluded from this study. Among the five immunophenotypes, there were more C1 and C2 in the high-PRMPI subgroup, while more C3 in the low-PRMPI subgroup (Figures 6E,F).

**FIGURE 7:**
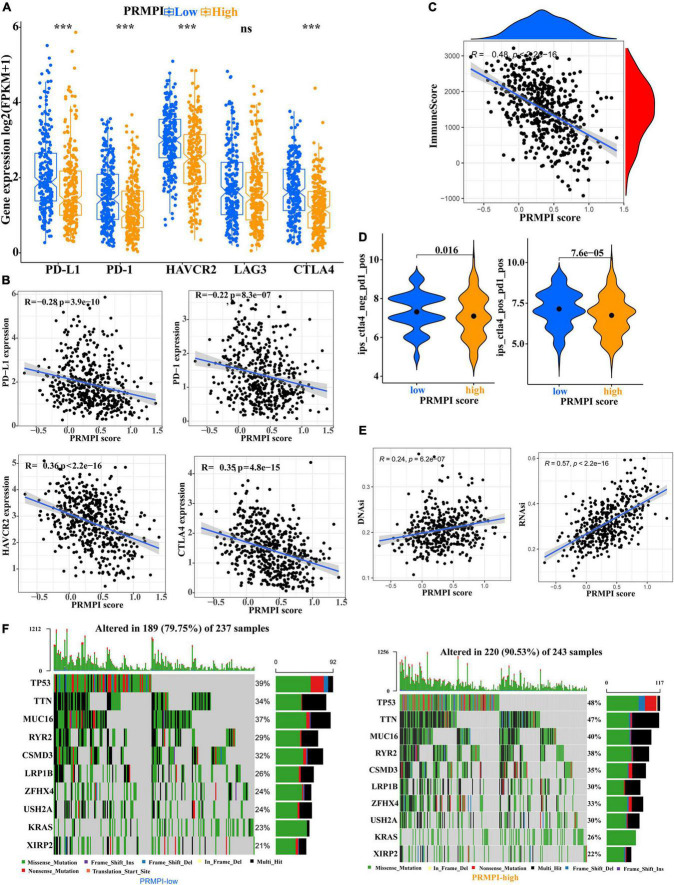
Levels of immune checkpoint molecule, immune score, relative probabilities to respond to immunotherapy, tumor stemness, and tumor mutation profile in the PRMPI subgroups. **(A)** Level of immune checkpoint molecules in low-PRMPI and high-PRMPI subgroups. Correlation analysis of the PRMPI score with immune checkpoint molecules **(B)** and immune scores **(C)**. **(D)** Relative probabilities to respond to immunotherapy in the low-PRMPI and high-PRMPI subgroups. **(E)** Relationship between the PRMPI score and DNAsi and RNAsi. **(F)** Mutation profile in the mutated LUAC samples from different PRMPI score subgroups. Mutated genes were sorted according to the mutation rate; the samples were arranged to exhibit mutual exclusivity between mutations.

Immune checkpoint molecules, such as PD-1, PD-L1, HAVCR2, LAG3, and CTLA-4, play an important role in tumor immune evasion. The expression level of these immune checkpoint molecules is an important reference indicator for individualized immunotherapy. In this study, except for LAG3, the expression levels of PD-1, PD-L1, HAVCR2, and CTLA-4 were significantly lower in the high-PRMPI subgroup than in the low-PRMPI subgroup (Figure 7A), and the expression levels of these immune checkpoint molecules were negatively correlated with the PRMPI score (Figure 7B). In addition, we have also explored the relationship between the PRMPI score and immune score, and the result showed that there is a significant negative correlation between them (Figure 7C).

The IPS is a score scheme based on machine learning, which can predict the response of patients with cancer to immunotherapy. Since there was no information on immunotherapy in the LUAD dataset, we used the IPS value as a substitute for the response of patients with LUAD to immunotherapy to verify our speculation. Interestingly, the result indicated that patients with LUAD generally had a better response to anti-PD-1 and anti-PD-1/CTLA-4 therapy in the low-PRMPI score subgroup (Figure 7D).

As there is a close relationship between tumor stemness and TIME (21), we reasonably speculate that the PRMPI scoring system constructed by PRMs may also be related to tumor stemness. Moreover, cancer stem cells play an important role in the TME, and we further analyze the relationship between tumor stem cells and PRMPI scores. Tumor stemness can be evaluated by RNAsi and DNAsi (21). The results showed that the PRMPI score was significantly positively associated with RNAsi and DNAsi (Figure 7E). The increase in tumor stemness is usually associated with tumor progression (21), which may be one of the reasons for the poor prognosis of the high-PRMPI score.

Next, we explored the gene mutations to get further biological insights into the pyroptosis characteristics of the different PRMPI subgroups. The result showed that the counts of gene mutations in the high-PRMPI subgroup were significantly higher than those in the low-PRMPI subgroup. The most common type of gene variation is a missense mutation, followed by multi-hit and nonsense mutations. Then, 10 genes with the highest variation rate in the high- and low-PRMPI groups were identified (Figure 7F). The variation rates of TP53, TTN, and MUC16 were higher than 40% in the high-PRMPI score subgroup, while these genes were less than 40% in the low-PRMPI subgroup. These results showed that there were significant differences in gene mutation rates between the high- and low-PRMPI subgroups.

### Analysis of the relationship between pyroptosis-related mRNA prognostic index score and efficacy of chemotherapy and targeted therapy

**FIGURE 8:**
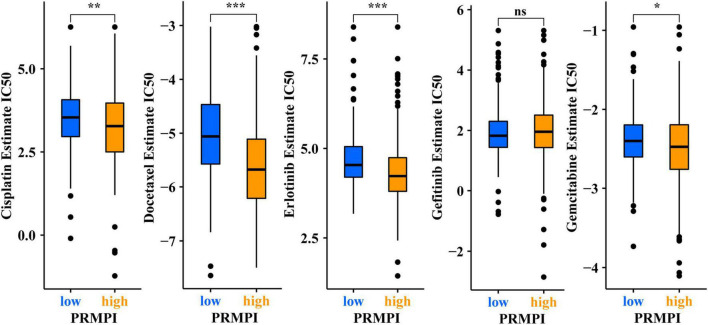
Pyroptosis-related mRNA prognostic index (PRMPI) may be a potential predictor of sensitivity to chemotherapy and targeted therapy, as high-PRMPI scores are associated with lower IC50 of targeted therapy and chemotherapy (*p-value < 0.05; **p-value < 0.01; and ***p-value < 0.001). ns, no significance.

In addition to immunotherapy, we also explored the association between PRMPI and the efficacy of first-line chemotherapy drugs and targeted drugs in treating LUAD in the TCGA cohort. The results showed that a high-PRMPI score was associated with a lower IC50 of chemotherapy drugs (cisplatin, docetaxel, and paclitaxel) and targeted drug (erlotinib), whereas it was not associated with targeted drug gefitinib (*p* > 0.05), which indicated that the PRMPI might be a potential predictor for chemotherapy and targeted therapy (Figure 8).

## Discussion

This study explored the expression pattern, clinical prognostic significance, relationship with the TIME, tumor stemness, and relationship with the tumor mutation state of PRMs in LUAD. The association between the PRMPI and the sensitivity of patients with LUAD to immunotherapy, chemotherapy, and targeted therapy was also explored. A total of five genes related to clinical prognostic significance were identified from 36 DEPRMs. Then, we constructed the PRMPI based on these genes, which effectively divided patients with LUAD into high- and low-PRMPI subgroups by the median PRMPI score. The PRMPI score was related to immune checkpoint molecules, immune cell levels, immune-related pathways, immune score, gene mutation, and tumor cell stemness. The PRMPI score can effectively predict the sensitivity of patients to immunotherapy, chemotherapy, and targeted therapy.

Pyroptosis is a form of PCD, which has different effects on tumors in different genetic backgrounds and tumor tissues. On the one hand, promoting the pyroptosis of tumor cells is conducive to inhibiting the progression of tumors (12, 25, 26). On the other hand, as a kind of pro-inflammatory PCD, pyroptosis can form a microenvironment suitable for tumor cell growth (7, 25, 27). In LUAD, it is unclear how pyroptosis-related molecules interact and whether they are associated with survival time. In this study, we constructed a PRMPI based on five PRMs (BAK1, CYCS, NLRC4, NLRP1, and NOD1) and found that it can effectively predict the OS of patients with LUAD. BAK (BAK1) protein is one of the important pro-apoptotic molecules needed for PCD and apoptosis induction in tumor cells. However, the expression levels of BAK genes and proteins in LUAD tissues are higher than those in paracancerous tissues, and the increased expression of BAK is associated with poor prognosis (28). This is consistent with our results. It may be that only the formation of a heterodimer or homolog of BAK can induce cell apoptosis (29). The activation of BAK/BAX can increase the permeability of the mitochondrial outer membrane, which will cause the release of cytochrome c (CYCS) from the mitochondria into the cytosol. Then, CYCS activates caspase-9 and caspase-3, which can further trigger apoptosis or pyroptosis (30). Moreover, BAK can mediate pyroptosis induced by chemotherapy *via* the BAK/BAX-caspase-3-GSDME pathway (30). NLRC4 and NLRP1 belong to the Nod-like receptor family, which can trigger pyroptosis in the canonical pathway by activating caspase-1 (31). However, the research on the function of NLRC4 in tumors is very limited. One study showed an anti-tumor effect in colon cancer, while another study showed that NLRC4 had no effect on cancer progression (32, 33). Recent research confirmed that NLRC4 plays a critical role in inhibiting the progression of melanoma, and lower expression levels of NLRC4 are an adverse factor in inhibiting tumor growth (34). The NLRC4 colon cancer mice model exhibited a greater increase in tumor burden than the WT mice model (32). The expression level of NLRP1 in colorectal cancer tissues is significantly lower than that in adjacent normal tissues, and NLRP1 can inhibit tumorigenesis of colorectal cancer (35). However, another study showed that the increase in NLRP1 expression and functional activity can promote the growth of melanoma (36). In our study, the expression levels of NLRC4 and NLRP1 in LUAD tissues were significantly lower than those in adjacent tissues, and the reduced expression levels significantly shortened the OS. The low expression of NLRP1 is associated with poor prognosis and a low level of tumor immune cell infiltration in patients with LUAD. However, there are few studies on NLRC4 and NLRP1 in lung cancer, which need to be further studied. Recent studies have shown that there are differences in the function of NOD1 in different types of tumors. In the triple-negative breast cancer model, reducing the expression of NOD1 can significantly inhibit the proliferation of tumor cells (37). Compared with normal liver tissues, the expression level of NOD1 in hepatocellular carcinoma tissues was significantly downregulated, and the increased expression level of NOD1 significantly inhibited the proliferation of tumor cells (38). By contrast, the expression level of NOD1 is significantly higher than that in adjacent tissues in colorectal cancer. The high expression of NOD1 is related to the poor prognosis of patients, and the activation of NOD1 promotes the metastasis of tumor cells (39). However, the role of NOD1 in lung cancer has not been studied. In our study, the expression level of NOD1 in LUAD tissues was significantly lower than that of adjacent tissues, and the increased expression of NOD1 was significantly related to the poor prognosis of patients. In summary, two genes (BAK1 and CYCS) from the PRMPI model were proven to be associated with an adverse prognosis, while another three genes (NLRC4, NLRP1, and NOD1) were identified as protective prognosis factors. How these five molecules interact with each other in the process of pyroptosis remains to be further explored.

Thus far, the research on pyroptosis in tumors is very limited, although certain crossovers in mechanisms and some similarities with apoptosis have been found. In the process of tumor formation and development, many types of cell death (such as apoptosis, pyroptosis, and necrosis) may coexist and influence each other (40). For example, the proteins encoded by five genes (BAK1, CYCS, NLRC4, NLRP1, and NOD1) are also key molecules in regulating apoptosis. However, apoptosis is characterized by an intact plasma membrane, no release of cell contents, and no inflammatory response, while the characteristic of pyroptosis is the opposite (41). The release of pyroptosis-related molecules IL-1β and IL-18 can induce the infiltration and activation of tumor immune cells, which is helpful to the anti-tumor immune response (42). We speculate that five pyroptosis-related mRNAs from the PRMPI model may regulate the components of TIME to some extent. Therefore, we extensively analyzed the relationship between the PRMPI and TIME, and the results showed that the level of anti-tumor-infiltrating immune cells in high-PRMPI patients was significantly lower than that of low-PRMPI subgroups, indicating that the immune function in the LUAD of high-PRMPI subgroups was suppressed. However, the level of infiltrating Treg cells in LUAD tissues in the low-PRMPI subgroup was significantly higher than that in the high-PRMPI group in the TCGA cohort, while it has been reported that Treg cells can inhibit anti-tumor immune response and are associated with poor prognosis in previous studies (43, 44). One possible factor for this is that it is essential for Treg cells to regulate the over-activated inflammatory response induced by pyroptosis in the TME. In addition, studies have shown that the two subtypes of Treg cells in colon cancer can promote anti-tumor immune response (45). The infiltration level of NK cells in LUAD was opposite to that in Treg cells. NK cells can inhibit the proliferation of lung cancer cells by producing cytokines, such as IFN-γ (46). Previous studies have shown that the infiltration level of NK cells in lung cancer tissues is positively correlated with the survival time of patients (47). However, other studies have proved that the tumoricidal ability to infiltrate NK cells in the TME is inhibited (48, 49). Therefore, it is of great significance to further study the regulatory mechanism of Treg and NK cells in LUAD. Similarly, the activation of most immune response pathways in the low-PRMPI subgroup was higher than that in the high-PRMPI subgroup in TCGA and CPTAC cohorts. Based on these findings, we speculated that the poor prognosis of high-PRMPI patients may be due to the decrease in anti-tumor immunity.

Recent studies have shown that the immune checkpoint molecule PD-L1 can switch the apoptosis of tumor cells to pyroptosis by mediating the expression of GSDMC (50). There may be a close relationship between the PRMPI and immune checkpoint molecules, such as PD-1, PD-L1, CTLA4, HAVCR2, and LAG3. Interestingly, we found that the expression levels of most of the immune checkpoint molecules in the low-PRMPI group were significantly higher than those in the low-PRMPI subgroup. In addition, these immune checkpoint genes and immune scores were negatively correlated with PRMPI scores. As such, low-PRMPI patients may get more benefits from immunotherapy than high-PRMPI patients. Therefore, we speculated that patients with LUAD with high-PRMPI scores are less sensitive to immunotherapy than those with low-PRMPI scores. The IPS is positively correlated with cancer immunogenicity and can predict the response of patients with cancer to immunotherapy (16). As we hypothesized, the result showed patients with LUAD who responded to immunotherapy showed a lower PRMPI score. Therefore, the PRMPI score may be an excellent predictor for immunotherapy in LUAD. In addition to immunotherapy, chemotherapy was also significantly associated with pyroptosis (51). We attempted to explore the relationship between the PRMPI score and chemotherapy and targeted therapy. We found that, unlike immunotherapy, chemotherapy and targeted therapy are generally more sensitive to patients with high PRMPI scores. It may be of great significance to adopt different treatment schemes according to different PRMPI scores for the LUAD individualized treatment.

In terms of immune subtypes, which were classified based on 160 immune expression signatures (18), our results revealed that the more aggressive immunophenotypes (C1 and C2) were more common in the high-PRMPI subgroup, while the less aggressive immunophenotype (C3) was more common in the low-PRMPI subgroup. Therefore, the low-PRMPI group was characterized by lower tumor invasiveness and active immune response, while the high-PRMPI group was characterized by higher tumor invasiveness and immune-suppressive response.

At present, the research has revealed the existence of CSCs, the “bugs,” with the ability to self-renew and metastasize. The tumor microenvironment, the “bed,” plays an essential role in supporting CSCs and may also facilitate tumor formation and progression (52). Moreover, there is a close relationship between tumor stemness and TIME (21). Therefore, to better understand the biological feature of PRMs in TME or TIME, we explored the relationship between the PRMPI score and CSCs. The result indicated that the PRMPI was positively correlated with DNAsi and RNAsi. This may be one of the reasons why patients with high PRMPI scores usually have poor prognoses.

To further understand how these five PRMs affect tumorigenesis, tumor progression, and the prognosis of patients with LUAD, we explored the gene mutation status of different PRMPI-score subgroups. The results showed that p53 and TTN gene mutations are the most common in both subgroups, and the mutation rate is higher in high-PRMPI samples than in low-PRMPI samples. P53 mutation is one of the most common genetic events in tumors, and it is also related to more tumor aggressiveness and poor prognosis in patients with cancer (53). Therefore, patients with LUAD with higher P53 mutations in the high-PRMPI subgroup have a worse prognosis than those with lower P53 mutations in the low-PRMPI score subgroup.

There are several limitations to our study. First, the function of PRMs in LUAD and the interaction between them have not been fully verified through *in vivo* and *in vitro* experiments. Second, for the lack of sufficient transcriptome data available in our own center, the PRMPI scoring system and the interaction between pyroptosis-related mRNAs and TIME or TME were not externally verified. In addition, it is necessary to further explore the regulatory mechanism of PRMs in TIME or TME to improve the efficacy of LUAD immunotherapy. However, these results may provide enlightenment for further study, mainly focusing on the potential mechanism of pyroptosis-related mRNA regulation.

## Data availability statement

The authors declare all data supporting the findings of this study were downloaded from the TCGA database (https://portal.gdc.cancer.gov/).

## Ethics statement

The studies involving human participants were reviewed and approved by the Ethics Committee in The Second Affiliated Hospital of Harbin Medical University. Written informed consent for participation was not required for this study in accordance with the national legislation and the institutional requirements.

## Author contributions

HL designed the research, analyzed the data, completed the work of qRT-PCR, and wrote the manuscript. XC participated in processing the data and sample collection. HW, BP, JW, and PZ collected the literature and analyzed the data. LZ designed the research and reviewed the manuscript. All authors approved it for publication.

## Footnotes

1
https://portal.gdc.cancer.gov

2
https://proteomics.cancer.gov/programs/cptac

## Supplementary material

Supplementary Figure 1 The flow chart of include and exclude patients.
