# Supplementary figures and images for: Identification of a prognostic index system and tumor immune infiltration characterization for lung adenocarcinoma based on mRNA molecular of pyroptosis
Source: Front Med (Lausanne). 2022 Sep 15;9:934835. doi: 10.3389/fmed.2022.934835 (PMC9520088; doi:10.3389/fmed.2022.934835)

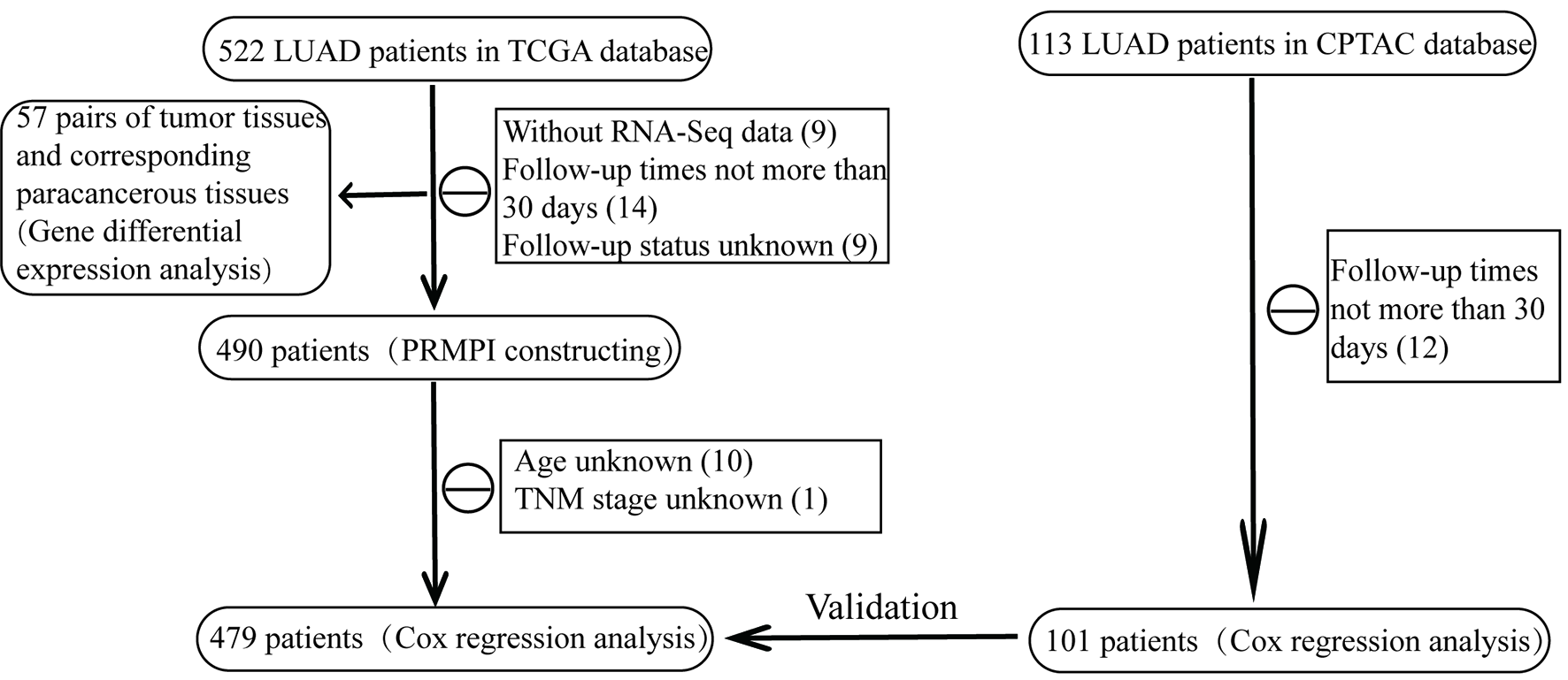

Supplement: Supplementary Figure 1 — The flow chart of include and exclude patients. [file Image_1.TIF]
