# Supplementary material for: Identification of a prognostic index system and tumor immune infiltration characterization for lung adenocarcinoma based on mRNA molecular of pyroptosis
Source: Front Med (Lausanne). 2022 Sep 15;9:934835. doi: 10.3389/fmed.2022.934835 (PMC9520088; doi:10.3389/fmed.2022.934835)
Supplement: Supplementary file 5 [file Table_4.DOC]

**Gene differential expression analysis**

library(limma)

inputFile="57-57.txt"

fdrFilter=0.05

logFCfilter=0

setwd("F:\\10.tcgaDiff")

rt=read.table(inputFile, header=T, sep="\t", check.names=F)

rt=as.matrix(rt)

rownames(rt)=rt[,1]

exp=rt[,2:ncol(rt)]

dimnames=list(rownames(exp), colnames(exp))

data=matrix(as.numeric(as.matrix(exp)), nrow=nrow(exp), dimnames=dimnames)

data=avereps(data)

data=data[rowMeans(data)>0,]

group=sapply(strsplit(colnames(data),"\\-"), "[", 4)

group=sapply(strsplit(group,""), "[", 1)

group=gsub("2", "1", group)

conNum=length(group[group==1])

treatNum=length(group[group==0])

Type=c(rep(1,conNum), rep(2,treatNum))

outTab=data.frame()

for(i in row.names(data)){

rt=data.frame(expression=data[i,], Type=Type)

wilcoxTest=wilcox.test(expression ~ Type, data=rt)

conGeneMeans=mean(data[i,1:conNum])

treatGeneMeans=mean(data[i,(conNum+1):ncol(data)])

logFC=log2(treatGeneMeans)-log2(conGeneMeans)

pvalue=wilcoxTest$p.value

conMed=median(data[i,1:conNum])

treatMed=median(data[i,(conNum+1):ncol(data)])

diffMed=treatMed-conMed

if( ((logFC>-8) & (diffMed>-8)) | ((logFC<0) & (diffMed<0)) ){

outTab=rbind(outTab,cbind(gene=i,conMean=conGeneMeans,treatMean=treatGeneMeans,logFC=logFC,pValue=pvalue))

}

}

pValue=outTab[,"pValue"]

fdr=p.adjust(as.numeric(as.vector(pValue)), method="fdr")

outTab=cbind(outTab, fdr=fdr)

write.table(outTab,file="tcga.all.xls",sep="\t",row.names=F,quote=F)

outDiff=outTab[( abs(as.numeric(as.vector(outTab$logFC)))>logFCfilter & as.numeric(as.vector(outTab$fdr))<fdrFilter),]

write.table(outDiff,file="tcga.diff.xls",sep="\t",row.names=F,quote=F)

write.table(outDiff,file="tcga.diff.txt",sep="\t",row.names=F,quote=F)

**Pheatmap and boxplot**

library(limma)

library(pheatmap)

library(reshape2)

library(ggpubr)

mRNAFile="interGenes.txt"

expFile="tcga.diffExp.txt"

setwd("F:\\14.pheatmap")

rt=read.table(expFile, header=T, sep="\t", check.names=F)

rt=as.matrix(rt)

rownames(rt)=rt[,1]

exp=rt[,2:ncol(rt)]

dimnames=list(rownames(exp),colnames(exp))

data=matrix(as.numeric(as.matrix(exp)),nrow=nrow(exp),dimnames=dimnames)

data=avereps(data)

data=data[rowMeans(data)>0,]

mRNA=read.table(mRNAFile, header=T, sep="\t", check.names=F, row.names=1)

data=data[colnames(mRNA)[1:ncol(mRNA)],]

data=log2(data+1)

exp=data

group=sapply(strsplit(colnames(data),"\\-"), "[", 4)

group=sapply(strsplit(group,""), "[", 1)

group=gsub("2", "1", group)

conNum=length(group[group==1])

treatNum=length(group[group==0])

sampleType=c(rep(1,conNum), rep(2,treatNum))

sigVec=c()

for(i in row.names(data)){

test=wilcox.test(data[i,] ~ sampleType)

pvalue=test$p.value

Sig=ifelse(pvalue<0.001,"***",ifelse(pvalue<0.01,"**",ifelse(pvalue<0.05,"*","")))

sigVec=c(sigVec, paste0(i, Sig))

}

row.names(data)=sigVec

Type=c(rep("Normal",conNum), rep("Tumor",treatNum))

names(Type)=colnames(data)

Type=as.data.frame(Type)

data=log2(data+0.01)

pdf("1heatmap.pdf", width=8, height=5)

pheatmap(data,

annotation=Type,

color = colorRampPalette(c(rep("#0066FF",5), "white", rep("#FF9900",5)))(100),

cluster_cols =F,

cluster_rows =T,

scale="row",

show_colnames=F,

show_rownames=T,

fontsize=10,

fontsize_row=10,

fontsize_col=10)

dev.off()

exp=as.data.frame(t(exp))

exp=cbind(exp, Type=sampleType)

exp$Type=ifelse(exp$Type==1, "Normal", "Tumor")

data=melt(exp, id.vars=c("Type"))

colnames(data)=c("Type", "Gene", "Expression")

p=ggboxplot(data, x="Gene", y="Expression", color = "Type",

ylab="Gene expression(FPKM)",

xlab="",

legend.title="Type",

palette = c("#0066FF", "#FF9900"),

notch = T,

width=0.55)

p=p+rotate_x_text(45)

p1=p+stat_compare_means(aes(group=Type),

method="wilcox.test",

symnum.args=list(cutpoints = c(0, 0.001, 0.01, 0.05, 1), symbols = c("***", "**", "*", " ")),

label = "p.signif")

pdf(file="boxplot.pdf", width=8, height=5)

print(p1)

dev.off()

**Forest**

library(survival)

coxFile="tcga.uniCox.txt"

geneFile="interGenes.txt"

setwd("F:\\15.forest")

rt=read.table(coxFile,header=T,sep="\t",row.names=1,check.names=F)

geneRT=read.table(geneFile,header=F,sep="\t",check.names=F)

rt=rt[as.vector(geneRT[,1]),]

gene=rownames(rt)

hr=sprintf("%.3f",rt$"HR")

hrLow=sprintf("%.3f",rt$"HR.95L")

hrHigh=sprintf("%.3f",rt$"HR.95H")

Hazard.ratio=paste0(hr,"(",hrLow,"-",hrHigh,")")

pVal=ifelse(rt$pvalue<0.001, "<0.001", sprintf("%.3f", rt$pvalue))

pdf(file="forest.pdf", width = 6,height = 4.3)

n=nrow(rt)

nRow=n+1

ylim=c(1,nRow)

layout(matrix(c(1,2),nc=2),width=c(3,2))

xlim = c(0,3)

par(mar=c(4,2.5,2,1))

plot(1,xlim=xlim,ylim=ylim,type="n",axes=F,xlab="",ylab="")

text.cex=0.8

text(0,n:1,gene,adj=0,cex=text.cex)

text(1.5-0.5*0.2,n:1,pVal,adj=1,cex=text.cex);text(1.5-0.5*0.2,n+1,'P-value',cex=text.cex,font=2,adj=1)

text(3,n:1,Hazard.ratio,adj=1,cex=text.cex);text(3,n+1,'Hazard Ratio',cex=text.cex,font=2,adj=1,)

par(mar=c(4,0,2,1),mgp=c(2,0.5,0))

xlim = c(0,max(as.numeric(hrLow),as.numeric(hrHigh)))

plot(1,xlim=xlim,ylim=ylim,type="n",axes=F,ylab="",xaxs="i",xlab="Hazard Ratio")

arrows(as.numeric(hrLow),n:1,as.numeric(hrHigh),n:1,angle=30,code=3,length=0.07,col="#6E568C",lwd=1.5)

abline(v=1,col="black",lty=3,lwd=3)

boxcolor = ifelse(as.numeric(hr) > 1, '#FF9900', '#0066FF')

points(as.numeric(hr), n:1, pch = 19, col = boxcolor, cex=1)

axis(1)

dev.off()

**LASSO**

library("Rcpp")

library("glmnet")

library("survival")

coxSigFile="tcga.uniSigExp.txt"

cptacFile="cptac.expTime.txt"

setwd("F:\\20.lasso")

rt=read.table(coxSigFile, header=T, sep="\t", check.names=F, row.names=1)

cptac=read.table(cptacFile, header=T, sep="\t", check.names=F, row.names=1)

sameGene=intersect(colnames(rt)[3:ncol(rt)], colnames(cptac)[3:ncol(cptac)])

rt=rt[,c("futime","fustat",sameGene)]

rt$futime=rt$futime/365

x=as.matrix(rt[,c(3:ncol(rt))])

y=data.matrix(Surv(rt$futime, rt$fustat))

fit=glmnet(x, y, family="cox", maxit=1000)

pdf("lasso.lambda.pdf")

plot(fit, xvar = "lambda", label = TRUE)

dev.off()

cvfit=cv.glmnet(x, y, family="cox", maxit=1000)

pdf("lasso.cvfit.pdf")

plot(cvfit)

abline(v=log(c(cvfit$lambda.min,cvfit$lambda.1se)),lty="dashed")

dev.off()

coef=coef(fit, s=cvfit$lambda.min)

index=which(coef != 0)

actCoef=coef[index]

lassoGene=row.names(coef)[index]

geneCoef=cbind(Gene=lassoGene, Coef=actCoef)

write.table(geneCoef, file="lasso.geneCoef.txt", sep="\t", quote=F, row.names=F)

trainFinalGeneExp=rt[,lassoGene]

myFun=function(x){crossprod(as.numeric(x),actCoef)}

trainScore=apply(trainFinalGeneExp,1,myFun)

outCol=c("futime","fustat",lassoGene)

risk=as.vector(ifelse(trainScore>median(trainScore),"high","low"))

outTab=cbind(rt[,outCol],PRMPI score=as.vector(trainScore),risk)

write.table(cbind(id=rownames(outTab),outTab),file="risk.TCGA.txt",sep="\t",quote=F,row.names=F)

rt=read.table(cptacFile, header=T, sep="\t", check.names=F, row.names=1)

rt$futime=rt$futime/365

testFinalGeneExp=rt[,lassoGene]

testScore=apply(testFinalGeneExp,1,myFun)

outCol=c("futime","fustat",lassoGene)

risk=as.vector(ifelse(testScore>median(trainScore),"high","low"))

outTab=cbind(rt[,outCol],PRMPI score=as.vector(testScore),risk)

write.table(cbind(id=rownames(outTab),outTab),file="risk.cptac.txt",sep="\t",quote=F,row.names=F)

**Survival**

library(survival)

library(survminer)

setwd("F:\\21.survival")

bioSurvival=function(inputFile=null, outFile=null){

rt=read.table(inputFile, header=T, sep="\t", check.names=F)

diff=survdiff(Surv(futime, fustat) ~risk,data = rt)

pValue=1-pchisq(diff$chisq,df=1)

pValue=signif(pValue,4)

pValue=format(pValue, scientific = TRUE)

fit <- survfit(Surv(futime, fustat) ~ risk, data = rt)

surPlot=ggsurvplot(fit,

data=rt,

conf.int=T,

pval=paste0("P=",pValue),

pval.size=5,

legend.title="PRMPI",

legend.labs=c("High-PRMPI", "Low-PRMPI"),

legend = c(0.8, 0.8),

font.legend=10,

xlab="Time(years)",

break.time.by = 2,

palette=c("#FF9900", "#0066FF"),

surv.median.line="hv",

risk.table=T,

risk.table.title="",

risk.table.height=.3)

pdf(file=outFile,onefile = FALSE,width = 5,height =4.5)

print(surPlot)

dev.off()

}

bioSurvival(inputFile="risk.TCGA.txt", outFile="tcga.survival.pdf")

bioSurvival(inputFile="risk.cptac.txt", outFile="cptac.survival.pdf")

**ROC**

library(survival)

library(survminer)

library(timeROC)

setwd("F:\\22.ROC")

bioROC=function(inputFile=null, rocFile=null){

rt=read.table(inputFile, header=T, sep="\t", check.names=F)

ROC_rt=timeROC(T=rt$futime,delta=rt$fustat,

marker=rt$PRMPIscore,cause=1,

weighting='aalen',

times=c(1,2,3),ROC=TRUE)

pdf(file=rocFile,width=5,height=5)

plot(ROC_rt,time=1,col='green',title=FALSE,lwd=2)

plot(ROC_rt,time=2,col='blue',add=TRUE,title=FALSE,lwd=2)

plot(ROC_rt,time=3,col='red',add=TRUE,title=FALSE,lwd=2)

legend('bottomright',

c(paste0('AUC at 1 years: ',sprintf("%.03f",ROC_rt$AUC[1])),

paste0('AUC at 2 years: ',sprintf("%.03f",ROC_rt$AUC[2])),

paste0('AUC at 3 years: ',sprintf("%.03f",ROC_rt$AUC[3]))),

col=c("green",'blue','red'),lwd=2,bty = 'n')

dev.off()

}

bioROC(inputFile="risk.TCGA.txt", rocFile="tcga.ROC.pdf")

bioROC(inputFile="risk.CPTAC.txt", rocFile="CPTAC.ROC.pdf")

**Risk Plot**

library(pheatmap)

setwd("F:\\23.riskPlot")

bioRiskPlot=function(inputFile=null, PRMPIscoreFile=null, survStatFile=null){

rt=read.table(inputFile,sep="\t",header=T,row.names=1,check.names=F)

rt=rt[order(rt$PRMPIscore),]

riskClass=rt[,"risk"]

lowLength=length(riskClass[riskClass=="low"])

highLength=length(riskClass[riskClass=="high"])

lowMax=max(rt$PRMPIscore[riskClass=="low"])

line=rt[,"PRMPIscore"]

line[line>10]=10

pdf(file=PRMPIscoreFile, width=6, height=4)

plot(line, type="p", pch=20,

xlab="Patients (increasing PRMPIsocre)", ylab="PRMPIscore",

col=c(rep("#0066FF",lowLength),rep("#FF9900",highLength)) )

abline(h=lowMax,v=lowLength,lty=2)

legend("topleft", c("High-PRMPI", "Low-PRMPI"),bty="n",pch=19,col=c("#FF9900","#0066FF"),cex=1.2)

dev.off()

color=as.vector(rt$fustat)

color[color==1]="#FF9900"

color[color==0]="#0066FF"

pdf(file=survStatFile, width=6, height=4)

plot(rt$futime, pch=19,

xlab="Patients (increasing PRMPIsocre)", ylab="Survival time (years)",

col=color)

legend("topleft", c("Dead", "Alive"),bty="n",pch=19,col=c("#FF9900","#0066FF"),cex=1.2)

abline(v=lowLength,lty=2)

dev.off()

}

bioRiskPlot(inputFile="risk.TCGA.txt",PRMPIscoreFile="tcga.PRMPIscore.pdf",survStatFile="tcga.survStat.pdf")

bioRiskPlot(inputFile="risk.CPTAC.txt",PRMPIscoreFile="CPTAC.PRMPIscore.pdf",survStatFile="CPTAC.survStat.pdf")

**PCA and t-SNE**

library(Rcpp)

library(Rtsne)

library(ggplot2)

setwd("F:\\24.PCA")

bioPCA=function(inputFile=null, pcaFile=null, tsneFile=null){

rt=read.table(inputFile, header=T, sep="\t", check.names=F, row.names=1)

data=rt[c(3:(ncol(rt)-2))]

risk=rt[,"risk"]

data.pca=prcomp(data, scale. = TRUE)

pcaPredict=predict(data.pca)

PCA = data.frame(PC1=pcaPredict[,1], PC2=pcaPredict[,2], risk=risk)

pdf(file=pcaFile, height=3.5, width=4.5)

p=ggplot(data = PCA, aes(PC1, PC2)) + geom_point(aes(color = risk)) +

scale_colour_manual(name="PRMPI", values =c("#FF9900", "#0066FF"))+

theme_bw()+

theme(plot.margin=unit(rep(1.5,4),'lines'))+

theme(panel.grid.major = element_blank(), panel.grid.minor = element_blank())

print(p)

dev.off()

tsneOut=Rtsne(data, dims=2, perplexity=10, verbose=F, max_iter=500,check_duplicates=F)

tsne=data.frame(tSNE1=tsneOut$Y[,1], tSNE2=tsneOut$Y[,2], risk=risk)

pdf(file=tsneFile, height=3.5, width=4.5)

p=ggplot(data = tsne, aes(tSNE1, tSNE2)) + geom_point(aes(color = risk)) +

scale_colour_manual(name="PRMPI", values =c("#FF9900", "#0066FF"))+

theme_bw()+

theme(plot.margin=unit(rep(1.5,4),'lines'))+

theme(panel.grid.major = element_blank(), panel.grid.minor = element_blank())

print(p)

dev.off()

}

bioPCA(inputFile="risk.TCGA.txt", pcaFile="tcga.PCA.pdf", tsneFile="tcga.t-SNE.pdf")

bioPCA(inputFile="risk.CPTAC.txt", pcaFile="CPTAC.PCA.pdf", tsneFile="CPTAC.t-SNE.pdf")

**Independent prognostic analysis of TCGA cohort**

library(survival)

setwd("F:\\25.TCGAindep")

risk=read.table("risk.TCGA.txt",header=T,sep="\t",check.names=F,row.names=1)

cli=read.table("clinical.txt",header=T,sep="\t",check.names=F,row.names=1)

sameSample=intersect(row.names(cli),row.names(risk))

risk=risk[sameSample,]

cli=cli[sameSample,]

rt=cbind(futime=risk[,1],fustat=risk[,2],cli,PRMPI=risk[,(ncol(risk))])

uniTab=data.frame()

for(i in colnames(rt[,3:ncol(rt)])){

cox <- coxph(Surv(futime, fustat) ~ rt[,i], data = rt)

coxSummary = summary(cox)

uniTab=rbind(uniTab,

cbind(id=i,

HR=coxSummary$conf.int[,"exp(coef)"],

HR.95L=coxSummary$conf.int[,"lower .95"],

HR.95H=coxSummary$conf.int[,"upper .95"],

pvalue=coxSummary$coefficients[,"Pr(>|z|)"])

)

}

write.table(uniTab,file="uniCox.txt",sep="\t",row.names=F,quote=F)

uniTab=uniTab[as.numeric(uniTab[,"pvalue"])<0.05,]

rt1=rt[,c("futime","fustat",as.vector(uniTab[,"id"]))]

multiCox=coxph(Surv(futime, fustat) ~ ., data = rt1)

multiCoxSum=summary(multiCox)

multiTab=data.frame()

multiTab=cbind(

HR=multiCoxSum$conf.int[,"exp(coef)"],

HR.95L=multiCoxSum$conf.int[,"lower .95"],

HR.95H=multiCoxSum$conf.int[,"upper .95"],

pvalue=multiCoxSum$coefficients[,"Pr(>|z|)"])

multiTab=cbind(id=row.names(multiTab),multiTab)

write.table(multiTab,file="multiCox.txt",sep="\t",row.names=F,quote=F)

bioForest=function(coxFile=null,forestFile=null,height=null){

rt <- read.table(coxFile,header=T,sep="\t",row.names=1,check.names=F)

gene <- rownames(rt)

hr <- sprintf("%.3f",rt$"HR")

hrLow <- sprintf("%.3f",rt$"HR.95L")

hrHigh <- sprintf("%.3f",rt$"HR.95H")

Hazard.ratio <- paste0(hr,"(",hrLow,"-",hrHigh,")")

pVal <- ifelse(rt$pvalue<0.001, "<0.001", sprintf("%.3f", rt$pvalue))

pdf(file=forestFile, width = 6.3,height = 4)

n <- nrow(rt)

nRow <- n+1

ylim <- c(1,nRow)

layout(matrix(c(1,2),nc=2),width=c(3,2.5))

xlim = c(0,3)

par(mar=c(4,2.5,2,1))

plot(1,xlim=xlim,ylim=ylim,type="n",axes=F,xlab="",ylab="")

text.cex=0.8

text(0,n:1,gene,adj=0,cex=text.cex)

text(1.5-0.5*0.2,n:1,pVal,adj=1,cex=text.cex);text(1.5-0.5*0.2,n+1,'P-value',cex=text.cex,adj=1)

text(3,n:1,Hazard.ratio,adj=1,cex=text.cex);text(3,n+1,'Hazard Ratio',cex=text.cex,adj=1,)

par(mar=c(4,1,2,1),mgp=c(2,0.5,0))

xlim = c(0,max(as.numeric(hrLow),as.numeric(hrHigh)))

plot(1,xlim=xlim,ylim=ylim,type="n",axes=F,ylab="",xaxs="i",xlab="Hazard Ratio")

arrows(as.numeric(hrLow),n:1,as.numeric(hrHigh),n:1,angle=30,code=3,length=0.05,col="#6E568C",lwd=1.5)

abline(v=1,col="black",lty=3,lwd=3)

boxcolor = ifelse(as.numeric(hr) > 1, "#FF9900", "#0066FF")

points(as.numeric(hr), n:1, pch = 19, col = boxcolor, cex=1)

axis(1)

dev.off()

}

bioForest(coxFile="uniCox.txt",forestFile="uniForest.pdf",height=4.5)

bioForest(coxFile="multiCox.txt",forestFile="multiForest.pdf",height=3.5)

**Independent prognostic analysis of CPTAC cohort**

library(survival)

setwd("F:\\25.CPTACAindep") risk=read.table("risk.CPTAC.txt",header=T,sep="\t",check.names=F,row.names=1) cli=read.table("clinical.txt",header=T,sep="\t",check.names=F,row.names=1)

sameSample=intersect(row.names(cli),row.names(risk))

risk=risk[sameSample,]

cli=cli[sameSample,]

rt=cbind(futime=risk[,1],fustat=risk[,2],cli,PRMPI=risk[,(ncol(risk))])

uniTab=data.frame()

for(i in colnames(rt[,3:ncol(rt)])){

cox <- coxph(Surv(futime, fustat) ~ rt[,i], data = rt)

coxSummary = summary(cox)

uniTab=rbind(uniTab,

cbind(id=i,

HR=coxSummary$conf.int[,"exp(coef)"],

HR.95L=coxSummary$conf.int[,"lower .95"],

HR.95H=coxSummary$conf.int[,"upper .95"],

pvalue=coxSummary$coefficients[,"Pr(>|z|)"])

)

}

write.table(uniTab,file="uniCox.txt",sep="\t",row.names=F,quote=F)

uniTab=uniTab[as.numeric(uniTab[,"pvalue"])<0.05,]

rt1=rt[,c("futime","fustat",as.vector(uniTab[,"id"]))]

multiCox=coxph(Surv(futime, fustat) ~ ., data = rt1)

multiCoxSum=summary(multiCox)

multiTab=data.frame()

multiTab=cbind(

HR=multiCoxSum$conf.int[,"exp(coef)"],

HR.95L=multiCoxSum$conf.int[,"lower .95"],

HR.95H=multiCoxSum$conf.int[,"upper .95"],

pvalue=multiCoxSum$coefficients[,"Pr(>|z|)"])

multiTab=cbind(id=row.names(multiTab),multiTab)

write.table(multiTab,file="multiCox.txt",sep="\t",row.names=F,quote=F)

bioForest=function(coxFile=null,forestFile=null,height=NULL){

rt <- read.table(coxFile,header=T,sep="\t",row.names=1,check.names=F)

gene <- rownames(rt)

hr <- sprintf("%.3f",rt$"HR")

hrLow <- sprintf("%.3f",rt$"HR.95L")

hrHigh <- sprintf("%.3f",rt$"HR.95H")

Hazard.ratio <- paste0(hr,"(",hrLow,"-",hrHigh,")")

pVal <- ifelse(rt$pvalue<0.001, "<0.001", sprintf("%.3f", rt$pvalue))

pdf(file=forestFile, width = 6.3,height = 3)

n <- nrow(rt)

nRow <- n+1

ylim <- c(1,nRow)

layout(matrix(c(1,2),nc=2),width=c(3,2.5))

xlim = c(0,3)

par(mar=c(4,2.5,2,1))

plot(1,xlim=xlim,ylim=ylim,type="n",axes=F,xlab="",ylab="")

text.cex=0.8

text(0,n:1,gene,adj=0,cex=text.cex)

text(1.5-0.5*0.2,n:1,pVal,adj=1,cex=text.cex);text(1.5-0.5*0.2,n+1,'P-value',cex=text.cex,adj=1)

text(3,n:1,Hazard.ratio,adj=1,cex=text.cex);text(3,n+1,'Hazard Ratio',cex=text.cex,adj=1,)

par(mar=c(4,1,2,1),mgp=c(2,0.5,0))

xlim = c(0,max(as.numeric(hrLow),as.numeric(hrHigh)))

plot(1,xlim=xlim,ylim=ylim,type="n",axes=F,ylab="",xaxs="i",xlab="Hazard Ratio")

arrows(as.numeric(hrLow),n:1,as.numeric(hrHigh),n:1,angle=30,code=3,length=0.05,col="#6E568C",lwd=1.5)

abline(v=1,col="black",lty=3,lwd=3)

boxcolor = ifelse(as.numeric(hr) > 1, "#FF9900", "#0066FF")

points(as.numeric(hr), n:1, pch = 19, col = boxcolor, cex=1)

axis(1)

dev.off()

}

bioForest(coxFile="uniCox.txt",forestFile="uniForest.pdf",height=4.5)

bioForest(coxFile="multiCox.txt",forestFile="multiForest.pdf",height=3.5)

**ssGSEA**

library(limma)

library(ggpubr)

library(reshape2)

setwd("F:\\29.scoreCor")

scoreCor=function(riskFile=null, scoreFile=null, project=null){

data=read.table(scoreFile, header=T, sep="\t", check.names=F, row.names=1)

group=sapply(strsplit(colnames(data),"\\-"), "[", 4)

group=sapply(strsplit(group,""), "[", 1)

group=gsub("2", "1", group)

data=t(data[,group==0])

if(project=="TCGA"){

rownames(data)=gsub("(.*?)\\-(.*?)\\-(.*?)\\-(.*?)\\-.*", "\\1\\-\\2\\-\\3", rownames(data)) }

if(project=="CPTAC"){

rownames(data)=gsub("(.*?)\\-(.*?)\\-.*", "\\2", rownames(data)) }

data=avereps(data)

risk=read.table(riskFile,header=T,sep="\t",row.names=1,check.names=F)

sameSample=intersect(row.names(data),row.names(risk))

data=data[sameSample,,drop=F]

risk=risk[sameSample,,drop=F]

rt=cbind(data,risk[,c("PRMPIScore","risk")])

rt=rt[,-(ncol(rt)-1)]

immCell=c("aDCs","B_cells","CD8+_T_cells","DCs","iDCs","Macrophages",

"Mast_cells","Neutrophils","NK_cells","pDCs","T_helper_cells",

"Tfh","Th1_cells","Th2_cells","TIL","Treg")

rt1=rt[,c(immCell,"risk")]

data=melt(rt1,id.vars=c("risk"))

colnames(data)=c("Risk","Type","Score")

data$Risk=factor(data$Risk, levels=c("low","high"))

p=ggboxplot(data, x="Type", y="Score", color = "Risk",

ylab="PRMPI Score",add = "none",xlab="",palette = c("#0066FF","#FF9900"),

notch = T,

short.panel.labs=T,

size = 0.2,

width=0.6)

p=p+rotate_x_text(45)

p=p+stat_compare_means(aes(group=Risk),symnum.args=list(cutpoints = c(0, 0.001, 0.01, 0.05, 1), symbols = c("***", "**", "*", "ns")),label = "p.signif")

pdf(file=paste0(project,".immCell.pdf"), width=5, height=5)

print(p)

dev.off()

immFunction=c("APC_co_inhibition","APC_co_stimulation","CCR",

"Check-point","Cytolytic_activity","HLA","Inflammation-promoting",

"MHC_class_I","Parainflammation","T_cell_co-inhibition",

"T_cell_co-stimulation","Type_I_IFN_Reponse","Type_II_IFN_Reponse")

rt1=rt[,c(immFunction,"risk")]

data=melt(rt1,id.vars=c("risk"))

colnames(data)=c("Risk","Type","Score")

data$Risk=factor(data$Risk, levels=c("low","high"))

p=ggboxplot(data, x="Type", y="Score", color = "Risk",

ylab="Score",add = "none",xlab="",palette = c("#0066FF","#FF9900"),

notch = T,

short.panel.labs=T,

size = 0.2,

width=0.5)

p=p+rotate_x_text(60)

p=p+stat_compare_means(aes(group=Risk),symnum.args=list(cutpoints = c(0, 0.001, 0.01, 0.05, 1), symbols = c("***", "**", "*", "ns")),label = "p.signif")

pdf(file=paste0(project,".immFunction.pdf"), width=5, height=5)

print(p)

dev.off()

}

scoreCor(riskFile="risk.TCGA.txt", scoreFile="immScore.TCGA.txt", project="TCGA")

scoreCor(riskFile="risk.CPTAC.txt", scoreFile="1.txt", project="CPTAC")

**Immune Subtype**

library(limma)

library(ggpubr)

riskFile="risk.TCGA.txt"

subtypeFile="Subtype_Immune_Model_Based.txt"

setwd("F:\\30.immSubtype")

risk=read.table(riskFile, header=T, sep="\t", check.names=F, row.names=1)

subtype=read.table(subtypeFile, header=T, sep="\t", check.names=F, row.names=1)

rownames(subtype)=gsub("(.*?)\\-(.*?)\\-(.*?)\\-.*", "\\1\\-\\2\\-\\3", rownames(subtype))

sameSample=intersect(row.names(subtype), row.names(risk))

subtype=subtype[sameSample,]

subtype=gsub(".+Immune |\\)","",subtype)

risk=risk[sameSample,]

data=cbind(as.data.frame(risk), subtype)

typeTab=table(data$subtype)

typeName=names(typeTab[typeTab>3])

data=data[which(data[,"subtype"] %in% typeName),]

group=levels(factor(data$subtype))

data$subtype=factor(data$subtype, levels=group)

comp=combn(group,2)

my_comparisons=list()

for(i in 1:ncol(comp)){my_comparisons[[i]]<-comp[,i]}

boxplot=ggboxplot(data, x="subtype", y="PRMPIScore", color="subtype",

xlab="Immune subtype",

ylab="score",

legend.title="",

size = 0.1,

notch = T,

width=0.6,

short.panel.labs=F,

outlier.shape = 25,

bxp.errorbar= T,

add = "jitter")+

stat_compare_means(comparisons = my_comparisons)

pdf(file="immSubtype.pdf", width=4.5, height=5)

print(boxplot)

dev.off()

**Cell Stemness**

library(limma)

library(ggplot2)

library(ggpubr)

library(ggExtra)

riskFile="risk.TCGA.txt"

RNAssFile="StemnessScores_RNAexp_20170127.2.tsv"

DNAssFile="StemnessScores_DNAmeth_20170210.tsv"

setwd("F:\\126inflammatory\\31.Stemness")

risk=read.table(riskFile, header=T, sep="\t", check.names=F, row.names=1)

RNAss=read.table(RNAssFile, header=T, sep="\t",check.names=F, row.names=1)

RNAss=t(RNAss[1,,drop=F])

rownames(RNAss)=gsub("(.*?)\\-(.*?)\\-(.*?)\\-.*", "\\1\\-\\2\\-\\3", rownames(RNAss))

RNAss=avereps(RNAss)

DNAss=read.table(DNAssFile, header=T, sep="\t", check.names=F, row.names=1)

DNAss=t(DNAss[1,,drop=F])

rownames(DNAss)=gsub("(.*?)\\-(.*?)\\-(.*?)\\-.*", "\\1\\-\\2\\-\\3", rownames(DNAss))

DNAss=avereps(DNAss)

sameSample=Reduce(intersect,list(row.names(risk),row.names(DNAss),row.names(RNAss)))

risk=risk[sameSample,"PRMPIScore",drop=F]

RNAss=RNAss[sameSample,,drop=F]

DNAss=DNAss[sameSample,,drop=F]

data=cbind(RNAss, DNAss, risk)

xlab="PRMPIScore"

ylab="RNAss"

outFile="RNAss.cor.pdf"

x=as.numeric(data[,xlab])

y=as.numeric(data[,ylab])

df1=as.data.frame(cbind(x,y))

p1=ggplot(df1, aes(x, y)) +

xlab("PRMPI score") + ylab("RNAsi")+

geom_point() + geom_smooth(method="lm",formula = y ~ x) + theme_bw()+

stat_cor(method = 'spearman', aes(x =x, y =y))

p2=ggMarginal(p1, type="density", xparams=list(fill = "#0066FF"), yparams=list(fill = "#FF0000"))

pdf(file=outFile, width=4.2, height=4)

print(p2)

dev.off()

xlab="PRMPIScore"

ylab="DNAss"

outFile="DNAss.cor.pdf"

x=as.numeric(data[,xlab])

y=as.numeric(data[,ylab])

df1=as.data.frame(cbind(x,y))

p1=ggplot(df1, aes(x, y)) +

xlab("PRMPI score") + ylab("DNAsi")+

geom_point() + geom_smooth(method="lm",formula = y ~ x) + theme_bw()+

stat_cor(method = 'spearman', aes(x =x, y =y))

p2=ggMarginal(p1, type="density", xparams=list(fill = "#0066FF"), yparams=list(fill = "#FF0000"))

pdf(file=outFile, width=4.2, height=4)

print(p2)

dev.off()

**ImmuneScore**

library(limma)

library(ggplot2)

library(ggpubr)

library(ggExtra)

riskFile="risk.TCGA.txt"

scoreFile="scores.txt"

setwd("F:\\126inflammatory\\33.estimateCor")

risk=read.table(riskFile, header=T, sep="\t", check.names=F, row.names=1)

score=read.table(scoreFile, header=T, sep="\t", check.names=F, row.names=1)

score=as.matrix(score)

row.names(score)=gsub("(.*?)\\-(.*?)\\-(.*?)\\-(.*?)\\-.*", "\\1\\-\\2\\-\\3", row.names(score))

score=avereps(score)

sameSample=intersect(row.names(risk), row.names(score))

risk=risk[sameSample,"PRMPIScore",drop=F]

score=score[sameSample,,drop=F]

data=cbind(score, risk)

pdf(file=outFile, width=4.2, height=4)

print(p2)

dev.off()

xlab="PRMPIScore"

ylab="ImmuneScore"

outFile="ImmuneScore.cor.pdf"

x=as.numeric(data[,xlab])

y=as.numeric(data[,ylab])

df1=as.data.frame(cbind(x,y))

p1=ggplot(df1, aes(x, y)) +

xlab("PRMPIS score") + ylab(ylab)+

geom_point() + geom_smooth(method="lm",formula = y ~ x) + theme_bw()+

stat_cor(method = 'spearman', aes(x =x, y =y))

p2=ggMarginal(p1, type="density", xparams=list(fill = "#0066FF"), yparams=list(fill = "#FF0000"))

pdf(file=outFile, width=4.2, height=4)

print(p2)

dev.off()

**Immune checkpoint molecules**

library(limma)

library(ggplot2)

library(ggpubr)

library(ggExtra)

gene="CD274"

showName="PD-L1"

setwd("F:\\34.riskGene")

riskGene=function(riskFile=null, expFile=null, project=null){

rt=read.table(expFile, header=T, sep="\t", check.names=F)

rt=as.matrix(rt)

rownames(rt)=rt[,1]

exp=rt[,2:ncol(rt)]

dimnames=list(rownames(exp), colnames(exp))

data=matrix(as.numeric(as.matrix(exp)), nrow=nrow(exp), dimnames=dimnames)

data=avereps(data)

data=data[rowMeans(data)>0,]

group=sapply(strsplit(colnames(data),"\\-"), "[", 4)

group=sapply(strsplit(group,""), "[", 1)

group=gsub("2", "1", group)

data=data[,group==0]

data=rbind(data, gene=data[gene,])

exp=t(data[c("gene",gene),])

row.names(exp)=gsub("(.*?)\\-(.*?)\\-(.*?)\\-.*", "\\1\\-\\2\\-\\3\\", row.names(exp))

exp=avereps(exp)

risk=read.table(riskFile, header=T, sep="\t", check.names=F, row.names=1)

sameSample=intersect(row.names(exp), row.names(risk))

exp=exp[sameSample,]

exp=log2(exp+1)

risk=risk[sameSample,]

data=cbind(as.data.frame(exp), as.data.frame(risk))

data$risk=ifelse(data$risk=="high", "High", "Low")

group=levels(factor(data$risk))

data$risk=factor(data$risk, levels=c("Low", "High"))

comp=combn(group,2)

my_comparisons=list()

for(i in 1:ncol(comp)){my_comparisons[[i]]<-comp[,i]}

boxplot=ggboxplot(data, x="risk", y="gene", color="risk",

xlab="PRMPIS",

ylab=paste0(showName, " expression"),

legend.title="",

palette = c("#0066FF", "#FF9900"),

notch = T,

width=0.6,

add = "jitter")+

stat_compare_means(comparisons = my_comparisons)

pdf(file=paste0(showName, ".boxplot.pdf"), width=2.5, height=4.5)

print(boxplot)

dev.off()

xlab="riskScore"

ylab=gene

x=as.numeric(data[,xlab])

y=as.numeric(data[,ylab])

df1=as.data.frame(cbind(x,y))

p1=ggplot(df1, aes(x, y)) +

xlab("PRMPIS score") + ylab(paste0(showName, " expression"))+

geom_point() + geom_smooth(method="lm",formula = y ~ x) + theme_bw()+

stat_cor(method = 'spearman', aes(x =x, y =y))

p2=ggMarginal(p1, type="density", xparams=list(fill = "#0066FF"), yparams=list(fill = "#FF0000"))

pdf(file=paste0(showName, ".cor.pdf"), width=4.2, height=4)

print(p2)

dev.off()

}

riskGene(riskFile="risk.TCGA.txt", expFile="M.txt", project="TCGA")

**IPS**

library(ggpubr)

tciaFile="TCIA.txt"

scoreFile="risk.TCGA.txt"

setwd("F:\\39.IPS")

ips=read.table(tciaFile, header=T, sep="\t", check.names=F, row.names=1)

score=read.table(scoreFile, header=T, sep="\t", check.names=F, row.names=1)

sameSample=intersect(row.names(ips), row.names(score))

ips=ips[sameSample, , drop=F]

score=score[sameSample, "risk", drop=F]

data=cbind(ips, score)

data$risk=factor(data$risk, levels=c("low", "high"))

group=levels(factor(data$risk))

comp=combn(group, 2)

my_comparisons=list()

for(i in 1:ncol(comp)){my_comparisons[[i]]<-comp[,i]}

for(i in colnames(data)[1:(ncol(data)-1)]){

rt=data[,c(i, "risk")]

colnames(rt)=c("IPS", "risk")

gg1=ggviolin(rt, x="risk", y="IPS", fill = "risk",

xlab="PRMPIS score", ylab=i,

legend.title="riskScore",

palette=c("#0066FF", "#FF9900"),

add = "mean",add.params = list(fill="white"))+

stat_compare_means(comparisons = my_comparisons)

method="wilcox.test"

stat_compare_means(comparisons = my_comparisons,symnum.args=list(cutpoints = c(0, 0.001, 0.01, 0.05, 1), symbols = c("p<0.001", "**", "*", "ns")),label = "p.signif")

pdf(file=paste0(i, ".pdf"), width=3, height=4)

print(gg1)

dev.off()

}

**Analysis of chemotherapy drugs**

#####Drug list######

#A.443654, A.770041, ABT.263, ABT.888, AG.014699, AICAR, AKT.inhibitor.VIII, AMG.706, AP.24534,

#AS601245, ATRA, AUY922, Axitinib, AZ628, AZD.0530, AZD.2281, AZD6244, AZD6482, AZD7762, AZD8055,

#BAY.61.3606, Bexarotene, BI.2536, BIBW2992, Bicalutamide, BI.D1870, BIRB.0796, Bleomycin,

#BMS.509744, BMS.536924, BMS.708163, BMS.754807, Bortezomib, Bosutinib, Bryostatin.1, BX.795,

#Camptothecin, CCT007093, CCT018159, CEP.701, CGP.082996, CGP.60474, CHIR.99021, CI.1040, Cisplatin,

#CMK, Cyclopamine, Cytarabine, Dasatinib, DMOG, Docetaxel, Doxorubicin, EHT.1864, Elesclomol,

#Embelin, Epothilone.B, Erlotinib, Etoposide, FH535, FTI.277, GDC.0449, GDC0941, Gefitinib,

#Gemcitabine, GNF.2, GSK269962A, GSK.650394, GW.441756, GW843682X, Imatinib, IPA.3,

#JNJ.26854165, JNK.9L, JNK.Inhibitor.VIII, JW.7.52.1, KIN001.135, KU.55933, Lapatinib,

#Lenalidomide, LFM.A13, Metformin, Methotrexate, MG.132, Midostaurin, Mitomycin.C, MK.2206,

#MS.275, Nilotinib, NSC.87877, NU.7441, Nutlin.3a, NVP.BEZ235, NVP.TAE684, Obatoclax.Mesylate,

#OSI.906, PAC.1, Paclitaxel, Parthenolide, Pazopanib, PD.0325901, PD.0332991, PD.173074, PF.02341066,

#PF.4708671, PF.562271, PHA.665752, PLX4720, Pyrimethamine, QS11, Rapamycin, RDEA119, RO.3306,

#Roscovitine, Salubrinal, SB.216763, SB590885, Shikonin, SL.0101.1, Sorafenib, S.Trityl.L.cysteine,

#Sunitinib, Temsirolimus, Thapsigargin, Tipifarnib, TW.37, Vinblastine, Vinorelbine, Vorinostat,

#VX.680, VX.702, WH.4.023, WO2009093972, WZ.1.84, X17.AAG, X681640, XMD8.85, Z.LLNle.CHO, ZM.447439

library(limma)

library(ggpubr)

library(pRRophetic)

library(ggplot2)

set.seed(12345)

expFile="symbol.txt"

riskFile="risk.TCGA.txt"

drug="Paclitaxel"

setwd("F:\\47.pRRophetic")

rt = read.table(expFile, header=T, sep="\t", check.names=F)

rt=as.matrix(rt)

rownames(rt)=rt[,1]

exp=rt[,2:ncol(rt)]

dimnames=list(rownames(exp),colnames(exp))

data=matrix(as.numeric(as.matrix(exp)),nrow=nrow(exp),dimnames=dimnames)

data=avereps(data)

data=data[rowMeans(data)>0.5,]

group=sapply(strsplit(colnames(data),"\\-"), "[", 4)

group=sapply(strsplit(group,""), "[", 1)

group=gsub("2","1",group)

data=data[,group==0]

data=t(data)

rownames(data)=gsub("(.*?)\\-(.*?)\\-(.*?)\\-(.*)", "\\1\\-\\2\\-\\3", rownames(data))

data=avereps(data)

data=t(data)

senstivity=pRRopheticPredict(data, drug, selection=1)

senstivity=senstivity[senstivity!="NaN"]

senstivity[senstivity>quantile(senstivity,0.99)]=quantile(senstivity,0.99)

risk=read.table(riskFile, header=T, sep="\t", check.names=F, row.names=1)

sameSample=intersect(row.names(risk), names(senstivity))

risk=risk[sameSample, "risk",drop=F]

senstivity=senstivity[sameSample]

rt=cbind(risk, senstivity)

rt$risk=factor(rt$risk, levels=c("low", "high"))

type=levels(factor(rt[,"risk"]))

comp=combn(type, 2)

my_comparisons=list()

for(i in 1:ncol(comp)){my_comparisons[[i]]<-comp[,i]}

boxplot=ggboxplot(rt, x="risk", y="senstivity", color = "black", fill="risk", width = 0.4,

xlab="PRGPI",

ylab=paste0(drug, " Estimate IC50"),

legend.title='',

palette= c("#0066FF", "#FF9900"),

)+

stat_compare_means(comparisons=my_comparisons,symnum.args=list(cutpoints = c(0, 0.001, 0.01, 0.05, 1), symbols = c("***", "**", "*", "ns")),

label = "p.signif")

pdf(file=paste0(drug, ".pdf"), width=1.8, height=4.5)

print(boxplot)

dev.off()

**qRT-PCR**

library("ggpubr")

inputFile="NOD1.txt"

outFile="NOD1 pairDiff.pdf"

ylab="NOD1 expression"

setwd("F:\\68.pairDiff")

data=read.table(inputFile,sep="\t",header=T,check.names=F,row.names=1)

cond1=colnames(data)[1]

cond2=colnames(data)[2]

pdf(file=outFile, width=2.6, height=3.6)

ggpaired(data, cond1 = cond1, cond2 = cond2, fill = "condition", palette = c("#0066FF", "#FF9900"),width=0.35,

point.size = 0.4,line.size = 0.15,line.color = "#FFD121",linetype = "solid",short.panel.labs = F,

font.label = list(size = 6, color = "red"),repel = T,

label.rectangle = T,

legend.title="Type",xlab="",ylab = ylab)+

stat_compare_means(paired = T, label = "p.format", label.x = 1.35)

method="wilcox.test"

#stat_compare_means(paired = TRUE, symnum.args=list(cutpoints = c(0, 0.001, 0.01, 0.05, 1), symbols = c("***", "**", "*", "ns")),label = "p.signif",label.x = 1.35)

dev.off()
